# Supplementary material for: A Likelihood Approach for Real-Time Calibration of Stochastic Compartmental Epidemic Models
Source: PLoS Comput Biol. 2017 Jan 17;13(1):e1005257. doi: 10.1371/journal.pcbi.1005257 (PMC5240920; doi:10.1371/journal.pcbi.1005257)
Supplement: S1 File — (TAR.GZ) [file pcbi.1005257.s014.tar.gz › HSPH_Online-SI-Revision/MSS10/n100-severe/n100-severe_NEW_table8.pdf]

1	R0	{1.65278, 9.06793, 0.1}	{1.67732, 9.44073, 0.}	{{1.67732, 9.44073, 0.}, {1.67732, 9.44073, 0.}}	{1.4847, 4.11118, 100.}	inf	1287.	2862.08	{2814.21, 2904.8}	122.384	0.347064	newly	875.	1809.66	{1754., 1860.}	106.818	0.31549	Reff	0.583613	0.565859	{0.564945, 0.566645}	3.04211	ar	66 342.	69 611.	{69 434., 69 804.}	4.92748
2	R0	{1.4321, 3.80644, 0.1}	{1.43626, 3.41297, 3.84}	{{1.43579, 3.38828, 4.}, {1.43579, 3.38828, 4.}}	{0.290443, 10.7557, 3748.}	inf	47.	36.2447	{14.519, 55.3565}	32.335	0.198708	newly	65.	53.18	{21., 84.}	33.2	0.195967	Reff	0.65732	0.659383	{0.658892, 0.659506}	0.313929	ar	54 217.	54 157.9	{54 088., 54 219.}	0.128631
3	R0	{1.68388, 4.76028, 0.1}	{1.64953, 4.30394, 2.}	{{1.64953, 4.30394, 2.}, {1.64953, 4.30394, 2.}}	{2.03981, 9.58634, 1900.}	inf	45.	25.8507	{15.01, 34.8179}	42.7958	0.267175	newly	41.	28.96	{15., 41.}	31.8049	0.193179	Reff	0.534935	0.524339	{0.524172, 0.524509}	1.98076	ar	68 299.	68 242.1	{68 212., 68 275.}	0.0833687
4	R0	{1.66014, 6.95383, 1.}	{1.67405, 7.28757, 1.}	{{1.67405, 7.28757, 1.}, {1.67405, 7.28757, 1.}}	{0.837854, 4.79943, 0.}	inf	535.	1357.06	{1317., 1398.63}	153.657	0.40414	newly	463.	1059.15	{1021., 1100.}	128.758	0.359187	Reff	0.564215	0.555181	{0.554457, 0.555834}	1.60124	ar	66 820.	68 420.1	{68 275., 68 551.}	2.3946
5	R0	{1.69984, 11.2038, 1.}	{1.6082, 8.77064, 4.}	{{1.6082, 8.77064, 4.}, {1.6082, 8.77064, 4.}}	{5.39127, 21.7172, 300.}	inf	2209.	2852.3	{2806.63, 2894.93}	29.1218	0.110966	newly	1193.	1897.91	{1837., 1947.}	59.0872	0.201521	Reff	0.580631	0.538982	{0.538263, 0.53968}	7.17304	ar	68 462.	69 498.6	{69 306., 69 670.}	1.51411
6	R0	{1.65876, 6.66217, 1.}	{1.67405, 7.28757, 1.}	{{1.67405, 7.28757, 1.}, {1.67405, 7.28757, 1.}}	{0.921746, 9.3874, 0.}	inf	667.	1086.81	{1049.05, 1131.36}	62.9397	0.21181	newly	562.	847.72	{805., 893.}	50.8399	0.178191	Reff	0.560711	0.558851	{0.558004, 0.559475}	0.331626	ar	67 105.	67 938.2	{67 802., 68 112.}	1.2417
7	R0	{1.69966, 5.89996, 0.1}	{1.737, 5.43578, 0.}	{{1.737, 5.43578, 0.}, {1.737, 5.43578, 0.}}	{2.19683, 7.86755, 100.}	inf	268.	252.655	{228.711, 280.351}	8.21434	0.0374558	newly	239.	244.15	{213., 274.}	8.19665	0.0348505	Reff	0.52426	0.536044	{0.535556, 0.53645}	2.24767	ar	69 396.	69 429.8	{69 351., 69 518.}	0.0850193
8	R0	{1.53428, 6.3734, 0.1}	{1.54465, 5.30296, 4.}	{{1.54465, 5.30296, 4.}, {1.54465, 5.30296, 4.}}	{0.675927, 16.7955, 3900.}	inf	613.	372.436	{330.246, 411.305}	39.2438	0.218141	newly	560.	390.9	{338., 441.}	30.1964	0.158142	Reff	0.635852	0.643865	{0.643243, 0.644498}	1.26032	ar	59 855.	58 995.7	{58 836., 59 142.}	1.43572
9	R0	{1.58939, 3.48608, 0.1}	{1.54098, 3.31039, 5.}	{{1.54098, 3.31039, 5.}, {1.54098, 3.31039, 5.}}	{3.04587, 5.03976, 4900.}	inf	3.	13.3368	{3.6053, 22.5684}	348.431	0.591662	newly	4.	18.65	{6., 33.}	370.75	0.605864	Reff	0.594845	0.576568	{0.576425, 0.576699}	3.07265	ar	62 575.	62 601.8	{62 578., 62 632.}	0.0430204
10	R0	{1.58528, 5.5593, 0.1}	{1.59034, 5.09935, 4.}	{{1.59034, 5.09935, 4.}, {1.59034, 5.09935, 4.}}	{0.319359, 8.27355, 3900.}	inf	262.	181.836	{150.279, 220.392}	30.5971	0.162852	newly	240.	190.58	{152., 227.}	21.1583	0.107559	Reff	0.591785	0.59495	{0.594331, 0.595417}	0.53479	ar	63 039.	62 852.2	{62 766., 62 940.}	0.296388
11	R0	{1.58305, 4.62721, 0.1}	{1.61691, 4.41442, 4.}	{{1.61691, 4.41442, 4.}, {1.61691, 4.41442, 4.}}	{2.13889, 4.59872, 3900.}	inf	119.	106.617	{81.0088, 129.294}	15.4318	0.0757127	newly	130.	125.08	{94., 156.}	15.4	0.0718983	Reff	0.585079	0.597794	{0.597417, 0.598184}	2.17311	ar	63 222.	63 186.1	{63 108., 63 253.}	0.0877543
12	R0	{1.70234, 9.34626, 0.1}	{1.81389, 12.0711, 4.}	{{1.81389, 12.0711, 4.}, {1.81389, 12.0711, 4.}}	{6.55295, 29.154, 3900.}	inf	1435.	5101.12	{5054.3, 5149.99}	255.479	0.5508	newly	887.	2591.88	{2536., 2640.}	192.207	0.465627	Reff	0.572735	0.543767	{0.542852, 0.54459}	5.05791	ar	68 213.	75 036.3	{74 847., 75 251.}	10.0029
13	R0	{1.68556, 9.74954, 1.}	{1.67732, 9.44073, 0.}	{{1.67732, 9.44073, 0.}, {1.67732, 9.44073, 0.}}	{0.488924, 3.16745, 100.}	inf	1573.	1616.59	{1566.83, 1662.84}	3.01784	0.0128472	newly	954.	1021.99	{982., 1057.}	7.13732	0.0297523	Reff	0.561814	0.558336	{0.557554, 0.559133}	0.619058	ar	68 426.	68 620.5	{68 455., 68 778.}	0.300018
14	R0	{1.59876, 9.78897, 1.}	{1.56097, 8.77207, 2.}	{{1.56097, 8.77207, 2.}, {1.56097, 8.77207, 2.}}	{2.36362, 10.3883, 100.}	inf	2224.	2716.45	{2652.41, 2781.}	22.1426	0.0867977	newly	1422.	1864.74	{1807., 1914.}	31.135	0.117607	Reff	0.640335	0.617513	{0.616499, 0.6185}	3.56409	ar	63 553.	64 284.7	{64 034., 64 556.}	1.15134
15	R0	{1.43577, 5.12819, 1.}	{1.41571, 4.94504, 2.}	{{1.41571, 4.94504, 2.}, {1.41571, 4.94504, 2.}}	{1.39688, 3.57152, 100.}	inf	365.	330.284	{287.1, 373.024}	10.6983	0.050915	newly	410.	372.71	{321., 422.}	11.1585	0.0527929	Reff	0.682565	0.673522	{0.672917, 0.674132}	1.32488	ar	53 259.	53 092.6	{52 892., 53 255.}	0.338685
16	R0	{1.58866, 8.73191, 1.}	{1.61188, 8.7843, 3.}	{{1.61188, 8.7843, 3.}, {1.61188, 8.7843, 3.}}	{1.46151, 0.599927, 200.}	inf	1770.	875.536	{831.877, 916.291}	50.5347	0.306007	newly	1205.	603.8	{571., 638.}	49.8921	0.300535	Reff	0.609442	0.632766	{0.632102, 0.633465}	3.82723	ar	64 349.	62 196.4	{61 994., 62 365.}	3.34518
17	R0	{1.43012, 3.8163, 0.1}	{1.43289, 3.98572, 3.32}	{{1.42949, 4.00571, 0.}, {1.44762, 4.01966, 4.}}	{0.262038, 5.56097, 3254.}	inf	129.	132.483	{102.083, 164.229}	15.8481	0.0682358	newly	198.	175.27	{131., 220.}	16.6212	0.0823917	Reff	0.671055	0.672305	{0.670274, 0.679005}	0.270869	ar	53 353.	53 339.8	{53 219., 53 462.}	0.139655
18	R0	{1.47965, 5.16472, 0.1}	{1.47651, 5.4396, 3.}	{{1.47651, 5.4396, 3.}, {1.47651, 5.4396, 3.}}	{0.212245, 5.3223, 2900.}	inf	307.	404.164	{376.427, 433.394}	31.6494	0.118603	newly	352.	420.32	{390., 455.}	19.4318	0.0762124	Reff	0.658962	0.656129	{0.655695, 0.656534}	0.429956	ar	56 086.	56 323.	{56 153., 56 479.}	0.423029
19	R0	{1.57191, 5.2314, 0.1}	{1.59034, 5.09935, 4.}	{{1.59034, 5.09935, 4.}, {1.59034, 5.09935, 4.}}	{1.17263, 2.52421, 3900.}	inf	180.	135.053	{109.89, 156.424}	24.978	0.128235	newly	174.	141.83	{116., 166.}	18.8678	0.094507	Reff	0.584248	0.591813	{0.59147, 0.592159}	1.29498	ar	63 119.	62 986.2	{62 919., 63 062.}	0.214626
20	R0	{1.54435, 5.32911, 0.1}	{1.54465, 5.30296, 4.}	{{1.54465, 5.30296, 4.}, {1.54465, 5.30296, 4.}}	{0.0194651, 0.490783, 3900.}	inf	264.	186.66	{158.949, 212.437}	29.2956	0.153276	newly	266.	190.66	{161., 222.}	28.3233	0.147899	Reff	0.594389	0.5957	{0.595297, 0.596126}	0.220451	ar	61 870.	61 710.3	{61 633., 61 788.}	0.258995
21	R0	{1.57932, 4.90031, 1.}	{1.61691, 4.41442, 4.}	{{1.61691, 4.41442, 4.}, {1.61691, 4.41442, 4.}}	{2.38012, 9.91554, 300.}	inf	136.	118.046	{97.35, 140.919}	15.3028	0.0745916	newly	157.	137.38	{111., 169.}	16.1146	0.0775076	Reff	0.576847	0.590867	{0.590491, 0.591185}	2.43045	ar	63 595.	63 627.6	{63 554., 63 713.}	0.080651
22	R0	{1.47044, 4.07694, 1.}	{1.47472, 4.04546, 0.}	{{1.47488, 4.00571, 0.}, {1.47488, 4.06661, 0.}}	{0.321869, 0.772056, 100.}	inf	134.	133.24	{99.962, 163.407}	14.3077	0.0632469	newly	178.	172.73	{131., 217.}	15.3539	0.0696973	Reff	0.652831	0.654741	{0.654272, 0.655346}	0.323204	ar	55 895.	55 855.9	{55 742., 55 950.}	0.134413
23	R0	{1.64997, 3.34546, 0.1}	{1.65456, 3.37285, 0.}	{{1.65456, 3.37285, 0.}, {1.65456, 3.37285, 0.}}	{0.278209, 0.818631, 100.}	inf	4.	6.92999	{1.91851, 11.1533}	94.2516	0.288949	newly	5.	9.52	{3., 17.}	111.2	0.322925	Reff	0.554456	0.55595	{0.555876, 0.556031}	0.269466	ar	66 404.	66 408.8	{66 396., 66 424.}	0.0135082
24	R0	{1.67405, 4.50254, 0.1}	{1.64953, 4.30394, 2.}	{{1.64953, 4.30394, 2.}, {1.64953, 4.30394, 2.}}	{1.46459, 4.41077, 1900.}	inf	61.	46.4505	{28.7114, 60.2655}	26.0604	0.144552	newly	72.	53.06	{32., 72.}	28.6944	0.162825	Reff	0.536115	0.528503	{0.528235, 0.528757}	1.41982	ar	68 017.	68 012.3	{67 967., 68 053.}	0.038902
25	R0	{1.50663, 3.1286, 0.1}	{1.4804, 3.12918, 2.}	{{1.4804, 3.12918, 2.}, {1.4804, 3.12918, 2.}}	{1.74114, 0.0185167, 1900.}	inf	23.	24.8339	{9.60296, 40.6931}	42.0081	0.208775	newly	31.	36.8	{13., 59.}	47.4839	0.213647	Reff	0.620536	0.609704	{0.609469, 0.60993}	1.74552	ar	58 823.	58 852.	{58 812., 58 896.}	0.0584125
26	R0	{1.69822, 6.34992, 0.1}	{1.74092, 5.83416, 0.}	{{1.74092, 5.83416, 0.}, {1.74092, 5.83416, 0.}}	{2.5142, 8.1223, 100.}	inf	344.	287.765	{265.426, 310.269}	16.3474	0.0784284	newly	289.	263.67	{239., 290.}	9.6436	0.0448483	Reff	0.518704	0.532725	{0.532293, 0.533112}	2.70294	ar	69 816.	69 721.	{69 629., 69 803.}	0.145654
27	R0	{1.55869, 4.92988, 0.1}	{1.55618, 4.83753, 3.}	{{1.55618, 4.83753, 3.}, {1.55618, 4.83753, 3.}}	{0.161327, 1.8733, 2900.}	inf	156.	139.096	{114.95, 160.072}	12.9795	0.0625348	newly	170.	151.4	{123., 181.}	14.3176	0.0690451	Reff	0.591429	0.590738	{0.59038, 0.591106}	0.117237	ar	62 344.	62 242.7	{62 154., 62 335.}	0.172126
28	R0	{1.46085, 6.06899, 0.1}	{1.44485, 6.00596, 0.}	{{1.44485, 6.00596, 0.}, {1.44485, 6.00596, 0.}}	{1.09544, 1.0385, 100.}	inf	471.	402.522	{362.547, 438.969}	14.5388	0.0695809	newly	430.	387.62	{344., 435.}	10.5953	0.0500459	Reff	0.667083	0.660764	{0.660237, 0.661324}	0.947119	ar	55 484.	55 022.6	{54 862., 55 192.}	0.831609
29	R0	{1.63017, 6.25164, 0.1}	{1.65865, 5.3704, 5.}	{{1.65865, 5.3704, 5.}, {1.65865, 5.3704, 5.}}	{1.74711, 14.0962, 4900.}	inf	417.	122.492	{97.895, 146.005}	70.6255	0.537727	newly	364.	121.48	{95., 150.}	66.6264	0.483446	Reff	0.557469	0.572094	{0.571688, 0.572497}	2.62337	ar	66 231.	65 675.2	{65 600., 65 744.}	0.839139
30	R0	{1.45173, 5.68165, 0.1}	{1.454, 5.65591, 3.}	{{1.454, 5.65591, 3.}, {1.454, 5.65591, 3.}}	{0.156508, 0.452997, 2900.}	inf	490.	454.198	{409.78, 496.622}	8.00972	0.0369118	newly	473.	455.42	{407., 498.}	6.80338	0.0306636	Reff	0.663223	0.664781	{0.664161, 0.665418}	0.234998	ar	55 280.	55 146.1	{54 966., 55 310.}	0.278564
31	R0	{1.55808, 2.77316, 0.1}	{1.51531, 2.72346, 5.}	{{1.51531, 2.72346, 5.}, {1.51531, 2.72346, 5.}}	{2.74532, 1.79201, 4900.}	inf	8.	7.08894	{2.85245, 11.4577}	37.577	0.201889	newly	17.	11.83	{4., 22.}	43.7059	0.290582	Reff	0.608757	0.592059	{0.591989, 0.592122}	2.74305	ar	60 940.	60 941.2	{60 926., 60 962.}	0.0187069
32	R0	{1.56797, 3.90327, 0.1}	{1.53501, 4.02511, 0.}	{{1.53501, 4.02511, 0.}, {1.53501, 4.02511, 0.}}	{2.10217, 3.12138, 100.}	inf	58.	59.9342	{41.5306, 76.7855}	18.7569	0.0810922	newly	67.	73.61	{49., 96.}	23.1493	0.0954308	Reff	0.594323	0.5818	{0.58151, 0.582077}	2.10717	ar	62 228.	62 179.1	{62 126., 62 227.}	0.0886739
33	R0	{1.70027, 5.43261, 0.1}	{1.65865, 5.3704, 5.}	{{1.65865, 5.3704, 5.}, {1.65865, 5.3704, 5.}}	{2.44779, 1.14517, 4900.}	inf	114.	50.0977	{36.8229, 63.5545}	56.0546	0.367538	newly	106.	48.25	{33., 65.}	54.4811	0.355603	Reff	0.533137	0.521147	{0.520911, 0.521361}	2.24898	ar	68 798.	68 635.5	{68 600., 68 675.}	0.23617
34	R0	{1.61668, 6.86192, 0.1}	{1.65865, 5.3704, 5.}	{{1.65865, 5.3704, 5.}, {1.65865, 5.3704, 5.}}	{2.59612, 21.7362, 4900.}	inf	584.	269.576	{230.583, 306.593}	53.8397	0.338101	newly	504.	273.08	{234., 316.}	45.8175	0.268726	Reff	0.585885	0.60631	{0.60569, 0.606917}	3.48626	ar	64 693.	63 850.2	{63 732., 63 938.}	1.30271
35	R0	{1.55955, 3.45071, 0.1}	{1.54098, 3.31039, 5.}	{{1.54098, 3.31039, 5.}, {1.54098, 3.31039, 5.}}	{1.19078, 4.06641, 4900.}	inf	18.	25.7666	{11.707, 38.615}	58.1637	0.204217	newly	21.	36.02	{18., 57.}	80.4762	0.242748	Reff	0.596107	0.588889	{0.588686, 0.589105}	1.21086	ar	61 783.	61 817.7	{61 783., 61 855.}	0.0587378
36	R0	{1.65068, 4.91307, 0.1}	{1.64953, 4.30394, 2.}	{{1.64953, 4.30394, 2.}, {1.64953, 4.30394, 2.}}	{0.0695459, 12.3981, 1900.}	inf	177.	102.055	{79.0942, 121.905}	42.3417	0.244574	newly	195.	117.04	{91., 141.}	39.9795	0.22752	Reff	0.557286	0.558135	{0.557807, 0.558505}	0.152286	ar	66 453.	66 294.2	{66 233., 66 365.}	0.239011
37	R0	{1.46131, 3.06714, 0.1}	{1.4804, 3.12918, 2.}	{{1.4804, 3.12918, 2.}, {1.4804, 3.12918, 2.}}	{1.30619, 2.02271, 1900.}	inf	14.	26.1825	{9.54694, 39.133}	100.812	0.303771	newly	26.	40.26	{12., 61.}	74.0769	0.258094	Reff	0.636678	0.644814	{0.644622, 0.645058}	1.27786	ar	56 452.	56 489.6	{56 437., 56 539.}	0.0773046
38	R0	{1.71034, 5.2546, 0.1}	{1.737, 5.43578, 0.}	{{1.737, 5.43578, 0.}, {1.737, 5.43578, 0.}}	{1.55867, 3.44798, 100.}	inf	137.	234.884	{210.816, 258.809}	71.4479	0.232717	newly	136.	226.25	{201., 251.}	66.3603	0.2193	Reff	0.519003	0.525392	{0.524967, 0.525781}	1.23107	ar	69 816.	70 014.8	{69 940., 70 094.}	0.284763
39	R0	{1.43565, 5.57989, 1.}	{1.42659, 5.32185, 5.}	{{1.42659, 5.32185, 5.}, {1.42659, 5.32185, 5.}}	{0.631031, 4.62455, 400.}	inf	344.	281.478	{245.182, 319.018}	18.2711	0.0897563	newly	352.	298.82	{256., 345.}	15.4602	0.0754906	Reff	0.663615	0.660319	{0.65976, 0.660827}	0.496627	ar	54 407.	54 248.9	{54 117., 54 408.}	0.311486
40	R0	{1.40327, 4.00764, 0.1}	{1.40116, 4.24222, 4.}	{{1.40116, 4.24222, 4.}, {1.40116, 4.24222, 4.}}	{0.150217, 5.85334, 3900.}	inf	152.	173.052	{141.902, 203.337}	17.1491	0.0677683	newly	210.	221.08	{177., 264.}	13.3143	0.0559472	Reff	0.680319	0.679002	{0.678567, 0.679432}	0.193574	ar	51 976.	51 904.2	{51 763., 52 030.}	0.186817
41	R0	{1.63612, 4.26741, 0.1}	{1.61691, 4.41442, 4.}	{{1.61691, 4.41442, 4.}, {1.61691, 4.41442, 4.}}	{1.17414, 3.4449, 3900.}	inf	56.	56.4808	{40.6354, 73.7013}	18.3756	0.0821943	newly	65.	64.42	{42., 84.}	19.9077	0.0910385	Reff	0.573476	0.566735	{0.566448, 0.566989}	1.17549	ar	65 029.	65 023.7	{64 969., 65 077.}	0.0470098
42	R0	{1.57426, 6.50271, 0.1}	{1.58886, 7.29751, 1.}	{{1.58886, 7.29751, 1.}, {1.58886, 7.29751, 1.}}	{0.927701, 12.2226, 900.}	inf	569.	834.708	{799.939, 877.593}	46.6973	0.166151	newly	480.	665.57	{626., 709.}	38.6604	0.141466	Reff	0.602202	0.603567	{0.602885, 0.604113}	0.226651	ar	62 522.	63 220.1	{63 051., 63 377.}	1.11652
43	R0	{1.37818, 5.37636, 0.1}	{1.41571, 4.94504, 2.}	{{1.41571, 4.94504, 2.}, {1.41571, 4.94504, 2.}}	{2.72345, 8.02261, 1900.}	inf	520.	408.715	{356.599, 454.577}	21.401	0.106323	newly	563.	478.09	{413., 537.}	15.1137	0.0731694	Reff	0.701742	0.722429	{0.721754, 0.723082}	2.94796	ar	50 098.	50 024.8	{49 781., 50 242.}	0.310771
44	R0	{1.66606, 4.92641, 0.1}	{1.64953, 4.30394, 2.}	{{1.64953, 4.30394, 2.}, {1.64953, 4.30394, 2.}}	{0.99204, 12.6353, 1900.}	inf	140.	75.0752	{54.828, 93.7436}	46.3749	0.279667	newly	138.	86.11	{61., 107.}	37.6014	0.214535	Reff	0.551433	0.547033	{0.546723, 0.547364}	0.797827	ar	67 066.	66 924.1	{66 876., 66 969.}	0.211627
45	R0	{1.623, 5.9449, 0.1}	{1.65865, 5.3704, 5.}	{{1.65865, 5.3704, 5.}, {1.65865, 5.3704, 5.}}	{2.19661, 9.66379, 4900.}	inf	438.	407.85	{368.698, 450.333}	8.24654	0.0378659	newly	424.	412.44	{370., 445.}	6.45755	0.0288899	Reff	0.585189	0.598543	{0.597832, 0.59918}	2.28206	ar	64 554.	64 514.1	{64 373., 64 646.}	0.145723
46	R0	{1.65235, 5.51234, 0.1}	{1.65865, 5.3704, 5.}	{{1.65865, 5.3704, 5.}, {1.65865, 5.3704, 5.}}	{0.381332, 2.575, 4900.}	inf	274.	237.8	{206.17, 269.474}	13.9658	0.0670522	newly	254.	233.87	{199., 265.}	11.0591	0.0518161	Reff	0.558213	0.560942	{0.560359, 0.561437}	0.488895	ar	66 509.	66 482.	{66 390., 66 583.}	0.100077
47	R0	{1.62138, 8.82208, 0.1}	{1.67732, 9.44073, 0.}	{{1.67732, 9.44073, 0.}, {1.67732, 9.44073, 0.}}	{3.45008, 7.0125, 100.}	inf	1228.	1372.51	{1330.68, 1415.5}	11.7679	0.0482036	newly	834.	878.72	{839., 916.}	5.48921	0.0230052	Reff	0.585561	0.60334	{0.602593, 0.604031}	3.03614	ar	65 598.	65 970.6	{65 771., 66 198.}	0.573021
48	R0	{1.64073, 6.69261, 0.1}	{1.67405, 7.28757, 1.}	{{1.67405, 7.28757, 1.}, {1.67405, 7.28757, 1.}}	{2.03078, 8.88987, 900.}	inf	581.	1570.67	{1522.29, 1614.1}	170.338	0.431773	newly	486.	1227.82	{1173., 1279.}	152.638	0.402256	Reff	0.561064	0.55589	{0.555118, 0.556692}	0.922093	ar	66 438.	68 613.7	{68 442., 68 809.}	3.27474
49	R0	{1.65556, 3.796, 0.1}	{1.65456, 3.37285, 0.}	{{1.65456, 3.37285, 0.}, {1.65456, 3.37285, 0.}}	{0.0603803, 11.1473, 100.}	inf	19.	16.9499	{5.97899, 29.9249}	37.0761	0.208449	newly	19.	22.83	{6., 38.}	53.4211	0.223632	Reff	0.553586	0.553286	{0.553066, 0.553457}	0.054253	ar	66 575.	66 582.4	{66 554., 66 620.}	0.029831
50	R0	{1.56828, 3.65713, 0.1}	{1.56331, 3.72349, 2.}	{{1.56331, 3.72349, 2.}, {1.56331, 3.72349, 2.}}	{0.31709, 1.81443, 1900.}	inf	15.	24.4408	{9.24566, 37.3624}	77.6919	0.25822	newly	19.	32.14	{12., 50.}	84.8421	0.276785	Reff	0.584592	0.582591	{0.582386, 0.58282}	0.342336	ar	62 761.	62 768.1	{62 726., 62 808.}	0.0395787
